# Association between Postural Orthostatic Tachycardia Syndrome and Joint Hypermobility

**DOI:** 10.1155/2021/8875003

**Published:** 2021-04-28

**Authors:** Alexis Javier Atuesta-Rodriguez, Yimy F. Medina-Velasquez, Orfa Motta, Maria Isabel Narvaez-Reyes, Federico Rondon-Herrera

**Affiliations:** Rheumatology Unit, National University of Colombia, Colombia

## Abstract

Joint hypermobility syndrome refers to increased joint flexibility beyond the normal range of motion. This syndrome has a benign form known as Ehlers-Danlos syndrome type 3. This is a disorder in which hypermobility is accompanied by clinical manifestations in the absence of any systemic disease. A clinical finding associated with this condition is postural orthostatic tachycardia syndrome. The following is a rare case of joint hypermobility syndrome and postural orthostatic tachycardia syndrome. The relevance of this case report lies in the impact that this disease had on the patient's quality of life and the limitation in the performance of activities of daily living.

## 1. Introduction

Joint hypermobility syndrome (JHS) refers to increased joint flexibility beyond the normal range of motion. Benign joint hypermobility syndrome, also known as Ehlers-Danlos syndrome (EDS) type 3, is the presence of hypermobility accompanied by clinical manifestations in the absence of a systemic disease. It is clinically diagnosed based on the maneuvers described in the Beighton score and the Brighton diagnostic criteria and after ruling out other causes [1]. The following is the report of a rare case of JHS associated with postural orthostatic tachycardia syndrome (POTS). The relevance of this case report lies in the impact that this disease had on the patient's quality of life and the limitation in the performance of activities of daily living.

## 2. Clinical Case

This is the case of a 16-year-old female patient with a 4-year history of snapping sound in the joints of hips, elbows, and knees, especially in extension, with noninflammatory polyarthralgia and persistent headache. Her medical history included recurrent neurocardiogenic syncopal episodes since the age of 12, along with spontaneous recovery without sequelae.

**Figure 1:**
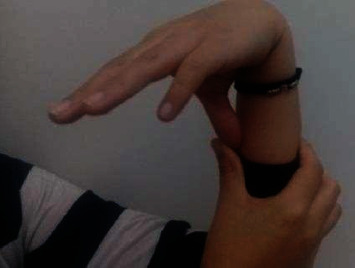
Hyperlaxity in the thumb.

**Figure 2:**
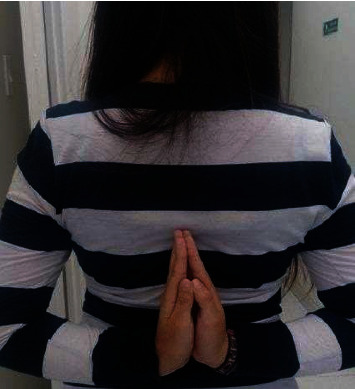
Hyperlaxity in the elbow.

**Figure 3:**
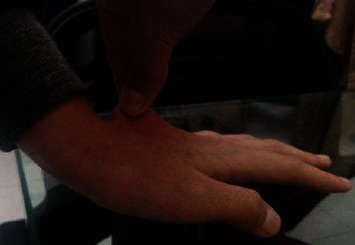
Cutaneous hyperlaxity.

**Figure 4:**
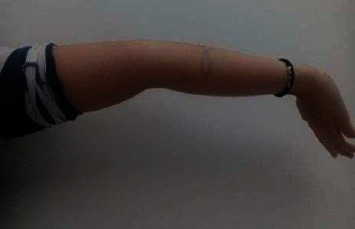
Hyperlaxity in the hand.

Physical examination showed joint hyperlaxity in hands, elbows, wrists, knees, and hips, with a Beighton score of 7/9 (Figures 1 and 2). Skin hyperlaxity was also observed (Figures 3 and 4). Blood pressure was 70/54 in decubitus position and 64/52 after sitting down for five minutes. Her heart rate (HR) was 80 beats per minute (bpm) when lying down and 110 bpm when sitting down. Elbow radiography showed radial head dislocation, while 24-hour Holter and blood pressure monitoring were normal. Tilt table test was compatible with mixed neurocardiogenic syncope and orthostatic intolerance syndrome.

Due to her history of neurocardiogenic syncope and joint hypermobility syndrome, after secondary causes were ruled out, she was diagnosed with JHS associated with orthostatic postural tachycardia syndrome (POTS). Nonpharmacological treatment was initiated, as well as increased fluid intake, sodium, postural therapy, among others. Clinical improvement was achieved due to a decrease in the frequency of syncopal episodes.

## 3. Discussion

POTS was initially described in 1993 by Schondorf and Low as a disorder of the autonomic nervous system characterized mainly by orthostatic intolerance [2, 3]. It is often underdiagnosed, and therefore, quality of life can be affected. Timely diagnostic and therapeutic approach can provide sufficient clinical control [4]. It occurs as a primary or secondary form of systemic diseases such as diabetes, joint hyperlaxity syndrome, paraneoplastic syndrome, among others [5]. POTS has a prevalence of 170 cases/100 000 inhabitants and is mainly observed in women between 20 and 40 years of age. It is caused by a failure of peripheral vascular resistance to orthostasis [6].

Although the definitions of POTS are strict, there are clinical variations in which not all the clinical findings mentioned above are observed, as in our patient's case. Some subtypes of POTS are classified considering whether the patient has a high, low, or normal flow. So, POTS can be neuropathic, hyperadrenergic, or autoimmune and may be associated with mast cell activation disorder and blood volume deregulation. None of these conditions is mutually exclusive or clearly independent [7].

Patients with POTS sometimes have a history of viral infection or a family member with similar symptoms; however, the actual association is unclear. The pathophysiology of this disease is mainly due to autonomic nervous system dysregulation, sympathetic hyperactivity, and attenuated vagal tone, resulting in hypoperfusion [8]. Clinical findings include cerebral hypoperfusion and sympathetic hyperactivity [3, 9]. Its diagnosis is based on clinical manifestations with different presentation frequencies (Table 1), such as an increase by 30 bpm compared to baseline HR vales or by 120 bpm when standing or walking within the first 10 minutes once orthostatic hypotension has been ruled out.

In the present case, although there was not a significant change in orthostasis, because there was not a decrease in systolic blood pressure (BP) of 20 mmHg or more or a decrease in diastolic BP of 10 mmHg or more, a significant variation in heart rate was indeed found when changing position. These symptoms should be present for at least six months and in the absence of another condition that could explain symptoms such as hydroelectrolytic alteration, secondary to drugs, among others. In our case, other causes were searched to explain the patient's clinical manifestations, but there were no other possible associations, and the symptoms had been present for more than 6 months.

Other findings that are not usually considered in this disease are high noradrenaline levels (600 ng/mL) and excessive response to isoproterenol. The differential diagnosis of POTS is pheochromocytoma due to sympathetic hyperreactivity, which causes neuropathies such as diabetes and vasovagal syncope [10].

Joint hyperlaxity syndrome (JHS) is characterized by joint hypermobility and other musculoskeletal manifestations in the absence of another disease. Men tend to have less hypermobility than women, and prevalence is usually inversely correlated to age, being higher in younger patients. However, there is confusion in terminology [3]. Type 3 or vascular EDS is associated with mutations in the COL3A1 allele. The main extra-articular clinical manifestations are fatigue, gastrointestinal disorders, neuropsychiatric, and cardiac manifestations, accompanied by increased skin elasticity and abnormal scarring. Musculoskeletal manifestations include polyarticular pain, enthesitis, bursitis, tenosynovitis, and mechanical lower back pain. Furthermore, a higher incidence of migraine, syncope, osteoarthritis, and osteoporosis has been found [10]. Pain is the most disabling symptom as it results from degeneration of ligaments and joint.

The association between POTS and JHS can be explained by predominant peripheral neuropathy in the autonomous system and a failure of the peripheral vascular system to maintain resistance in areas exposed to stress due to orthostasis, blood stasis in the lower limbs, or pathologies of the central nervous system [11]. The result is a functional decrease in blood volume with compensation given by increased heart rate and myocardia contractility. If compensation is not achieved in severe cases, cerebral hypoperfusion occurs with subsequent outcomes. Patients with postural orthostatic tachycardia and hypermobility syndrome present more symptoms from an early age [10].

The goal of treatment is based on nonpharmacological measures such as increased fluid intake, sodium, postural therapy, and rest, along with a multidisciplinary rehabilitative approach, which prevents muscle loss and deconditioning. Patients should perform physical activity 3 to 4 times per week for at least 45 minutes, achieving a frequency between 75 and 80% of the expected maximum heart rate with a progressive increase, mainly from aerobic exercise. Using elastic compression stockings in the lower limbs is also recommended with a pressure of at least 20 mmHg to improve venous return. Pharmacological therapy, such as the administration of fludrocortisone at doses of 0.05 to 0.2 mg per day, seeks to increase sodium retention to achieve blood volume expansion. Midodrine can also improve vascular resistance. Increased vagal tone with pyridostigmine may control postural orthostatic tachycardia, which is a similar mechanism obtained with the use of beta-blockers [3, 10, 12].

## 4. Conclusion

Orthostatic postural tachycardia syndrome associated with joint hypermobility is rare. The detection of patients with or susceptible to this disease becomes a challenge for clinicians to provide timely treatment that reduces complications and improves patients' quality of life.

## Figures and Tables

**Table 1:** Clinical manifestations and their relative frequencies in POTS.

Clinical manifestation	Frequency
Chronic fatigue	85-95%
Dizziness	60-80%
Palpitations	40-55%
Exercise intolerance	50-85%
Blurred vision	70%
Chest discomfort	60%
Headache	50%
Dyspnoea	40%
Syncope	40-50%

## Data Availability

We can get the data with the authors. There is a clinical record about the patient.
